# Emerging Viral Threats in Rice: A Decade of Discovery and Implications for Crop Protection

**DOI:** 10.1111/pce.70156

**Published:** 2025-09-02

**Authors:** Xinlun Ding, Feng Wang, Pingping Liu, Jie Zhang, Zujian Wu, Yan‐Hong Han, Jianguo Wu

**Affiliations:** ^1^ State Key Laboratory of Agricultural and Forestry Biosecurity Institute of Plant Virology, Vector‐Borne Virus Research Center, Fujian Agriculture and Forestry University Fuzhou China

**Keywords:** cultivated rice, high‐throughput sequencing, new viruses, wild rice

## Abstract

Rice viral diseases pose severe threats to global food security, with over 20 viruses identified in China alone. The advent of high‐throughput sequencing has accelerated the discovery of novel viruses in cultivated and wild rice, unveiling previously undetected threats. This review systematically summarises newly discovered rice viruses over the past decade, analyzing their genomic characteristics, transmission modes, and pathogenic mechanisms. Key findings include the identification of rice stripe mosaic virus, rice tiller inhibition virus (RTIV), RTIV2 and rice curl dwarf‐associated virus, among others, highlighting their interactions with host immunity and insect vectors. Notably, emerging viruses exhibit novel infection strategies, including interference with RNA silencing, hormone signalling, and autophagy pathways. Moreover, mixed infections and cross‐species transmission raise concerns about evolving disease dynamics. Understanding these viral threats is crucial for developing integrated disease management strategies, including resistant cultivars and vector control measures. This review provides a comprehensive resource for advancing rice virology research and guiding future efforts in monitoring and mitigating viral diseases affecting global rice production.

## Summary statement

• This review systematically summarises newly discovered rice viruses over the past decade, providing an in‐depth analysis of their genomic characteristics, transmission mechanisms, and interactions with host immunity and insect vectors.
• Our work synthesizes knowledge from molecular virology, genomics, and plant‐pathogen interactions, providing a valuable reference for researchers in plant disease resistance, crop protection, and sustainable agriculture.

## 1 Introduction

Rice is one of the most important food crops globally and serves as a staple grain crop in China. Rice viral diseases pose severe threats and are one of the important diseases in rice production, more than 20 types of rice viruses have been reported to date, among which the rice viruses commonly occurring in China include rice dwarf virus (RDV), rice gall dwarf virus (RGDV), rice grassy stunt virus (RGSV), rice ragged stunt virus (RRSV), rice stripe virus (RSV), rice black‐streaked dwarf virus (RBSDV), among others. A new rice disease, known as southern rice black‐streaked dwarf disease, was first discovered in Guangdong, China, in 2001 and was later confirmed to be caused by southern rice black‐streaked dwarf virus (SRBSDV) years later (Zhang et al. 2008; Zhou et al. 2008; Wang et al. 2010). The current research status on this disease has been extensively reviewed in multiple publications (Zhang and Zhou 2017; Chen et al. 2023; Wu et al. 2023).

The widespread application of high‐throughput sequencing (HTS) technologies in the field of plant virology has significantly accelerated the discovery rate of viruses. Particularly since 2017, multiple new viruses have been detected in both cultivated and wild rice in China and abroad. This paper systematically reviews the historical occurrence and research status of newly discovered viruses in *Oryza* species worldwide in recent years, thoroughly analyzes the main issues in current research, and discusses potential directions for future studies. This paper aims to serve as a valuable reference for advancing research on rice viral diseases and enhancing the monitoring and control in practical production settings.

## 2 Newly Discovered Viruses in *Oryza* in the Past Decade

### 2.1 Rice Stripe Mosaic Virus (RSMV)

RSMV, a member of the genus *Cytorhabdovirus* (family *Rhabdoviridae*), was first identified in Guangdong Province in 2015 (Yang, Zhang, et al. 2017). It has since spread to Guangdong, Guangxi, and Hainan, with a disease incidence of 5%–10% (Yang et al. 2018)., reaching over 40% in severe outbreaks (Yang et al. 2018; Chen et al. 2019a). Rice stripe mosaic disease caused by RSMV is characterised by pale yellow streaks or mosaic patterns on the leaves. In severe cases, leaf bases become twisted and deformed, panicle emergence is incomplete, and grains are poorly filled, leading to yield losses of approximately 50% (Yang, Huang, et al. 2017; Chen et al. 2019a; Chen et al. 2019b). Yield losses vary by rice cultivar, with *indica* rice (cv. Meixiangzhan) being the most severely affected, followed by hybrid *indica* (cv. Wuyou 1179) and *japonica* rice (Nipponbare) (Chen et al. 2019b).

**Figure 1:**
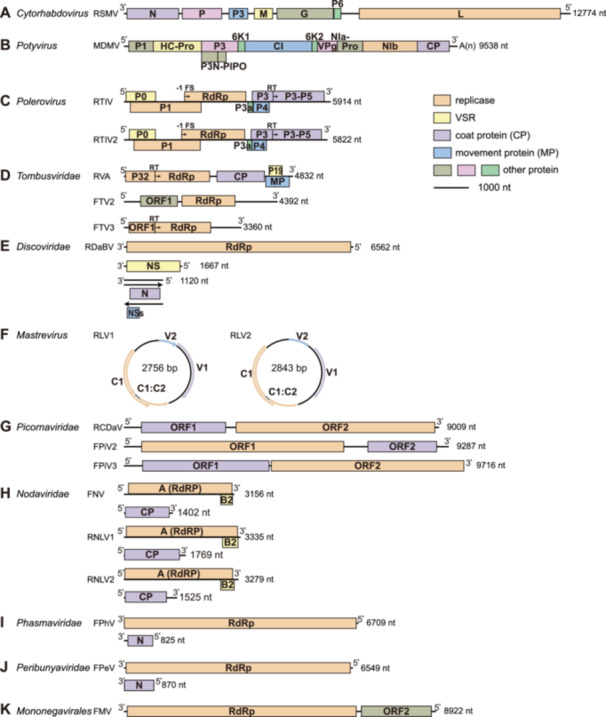
Genome organisation of viruses discussed in this article. (A) RSMV, rice stripe mosaic virus; (B) MDMV, maize dwarf mosaic virus; (C) RTIV, rice tiller inhibition virus; RTIV2, rice tiller inhibition virus 2; (D) RVA, rice virus A; FTV2, Fuyang tombus‐like virus 2; FTV3, Fuyang tombus‐like virus 3; (E) RDaBV, rice dwarf‐associated bunya‐like virus; (F) RLV1, rice latent virus 1; RLV2, rice latent virus 2; (G) RCDaV, rice curl dwarf‐associated virus; FPiV2, Fuyang picorna‐like virus 2; FPiV3, Fuyang picorna‐like virus 3; (H) FNV, Fuyang noda‐like virus; RNLV1, rice‐associated noda‐like virus 1; RNLV2, rice‐associated noda‐like virus 2; (I) FPhV, Fuyang phasma‐like virus; (J) FPeV, rice peribunya‐like virus; (K) FMV, Fuyang mononega‐like virus. Open reading frames (ORFs) encode proteins such as RNA‐dependent RNA polymerase (RdRp), coat protein (CP), movement protein (MP), viral suppressor of RNA silencing (VSR) and other functional proteins. These are shown in different colours for distinction. RSMV virion was modified from the *cytorhabdovirus* on Viralzone.

RSMV is transmitted in a persistent propagative manner by *Recilia dorsalis* (zigzag leafhopper) but not via eggs (Yang, Zhang, et al. 2017). *Nephotettix virescens* (green paddy leafhopper) acts as a secondary vector (Zhao et al. 2019). RSMV cannot be transmitted through seeds or mechanical wounding (Yang, Zhang, et al. 2017). Additionally, the weed *Digitaria sanguinalis* serves as a natural host of RSMV (Yang, Zhang, et al. 2017). RSMV is a rod‐shaped virus with particle dimensions of 300 × 55 nm and the viral particles are enveloped and exhibit 5.8 nm‐long spike‐like projections (Liu et al. 2019; Yang, Zhang, et al. 2017). The genome is a negative‐sense single‐stranded RNA (ssRNA) of 12 774 nucleotides, with 89 nt and 296 nt at the 3′ and 5′ termini, respectively. The genome encodes seven proteins: N, P, P3, M, G, P6 and L, of which P3 and P6 are nonstructural proteins with unclear functions (Figure 1A) (Yang et al. 2018; Yang, Zhang, et al. 2017). Thirteen RSMV genome sequences from South China (Guangdong, Guangxi and Yunnan) shared 99.4%–100% identities (Yang et al. 2018). RSMV has undergone strong purifying selection during evolution, with a transition rate of 72.08% and a transversion rate of 27.92%. Phylogenetic analysis based on whole‐genome nucleotide sequences revealed that RSMV isolates could be divided into two groups (Group I and Group II) based on geographic origin (Yang et al. 2018).

**Figure 2:**
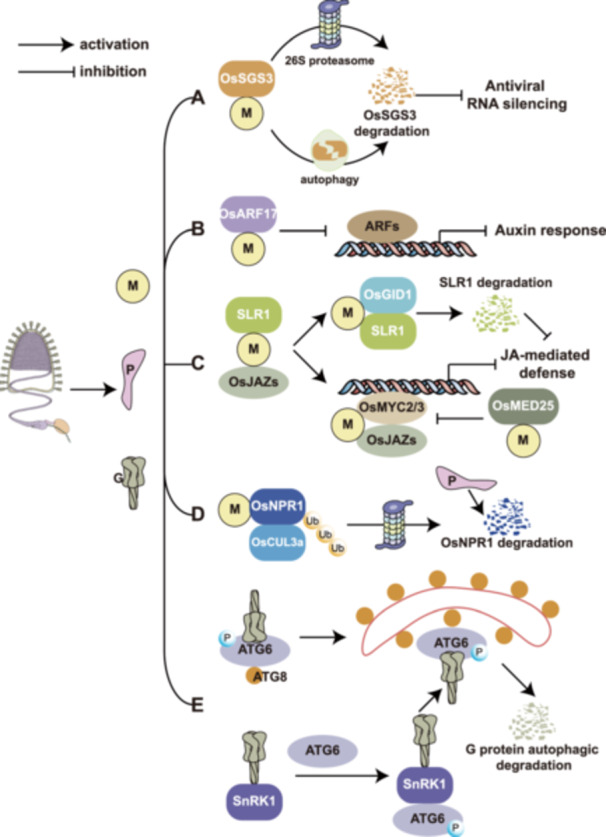
Mechanistic insights into rice stripe mosaic virus pathogenicity in rice. (A) The M protein interacting with OsSGS3 and promoting its degradation via ubiquitination and autophagy. (B) The M protein interacts with OsARF17 and suppresses its transcriptional activation function, inhibiting auxin‐signalling. (C) The M protein blocks the association of SLR1 and OsJAZ and promotes OsGID1 ‐SLR1 interaction, leading to SLR1 degradation and suppression of JA‐mediated resistance. The M protein negatively regulates the JA signalling pathway by inhibiting the interaction between OsJAZ, OsMYC2/3 and OsMED25. (D) The M protein enhances OsCUL3a mediated ubiquitination of OsNPR1, while the P protein promotes OsNPR1 degradation. (E) The G protein triggers host autophagy by interacting with SnRK1 and promotes its kinase activity on ATG6, and the G protein can be recognised by ATG6, which links the G protein to key ATG8 for degradation. [Color figure can be viewed at wileyonlinelibrary.com]

In recent years, significant progress has been made in understanding RSMV‐host interactions (Figure 2). The M protein of RSMV acts as a weak viral suppressors of RNA silencing (VSR) by interacting with the rice SGS3 protein (OsSGS3), a key component in the siRNA generation pathway, and promoting its degradation via ubiquitination and autophagy (Figure 2A) (C. Zhang et al. 2020). Transgenic rice expressing the M protein exhibited reduced plant height, increased tillering, and developmental defects, suggesting its involvement in viral pathogenicity. Additionally, the auxin‐signalling transcription factor OsARF17 plays a vital role in rice resistance to RSMV infection. OsARF17‐mediated antiviral immunity regulates the interplay among jasmonic acid (JA), auxin (IAA), and abscisic acid (ABA) pathways, modulating the expression of rice resistance genes (Ma et al. 2023). The M protein directly interacts with OsARF17 and suppresses its transcriptional activation function, weakening its antiviral defence and promoting viral infection (Figure 2B) (H. Zhang et al. 2020).

The M protein negatively regulates the JA signalling pathway by inhibiting the interaction between OsJAZ, OsMYC3 and OsMED25, facilitating viral infection and vector feeding (Figure 2C) (L. Li et al. 2021). Overexpression of the *SLR1* gene significantly enhances rice resistance to RSMV. It is hypothesised that SLR1 activates JA‐mediated antiviral responses through specific interactions with JA‐related components OsJAZ and OsMYC2/3 transcription factors. However, the M protein promotes the interaction between the GA receptor OsGID1 and SLR1, triggering SLR1 degradation and indirectly suppressing JA‐mediated antiviral immunity (Figure 2C) (Lulu Li et al. 2022). OsNPR1 plays a critical role in rice antiviral responses by interfering with the formation of the OsMYC2/3‐OsJAZ complex, activating the JA signalling pathway, and enhancing rice resistance. However, the P protein of RSMV promotes OsNPR1 degradation, impairing its antiviral function. Furthermore, the M protein enhances the interaction between OsNPR1 and OsCUL3a, mediating its ubiquitination and degradation, thereby disrupting the synergistic SA‐JA antiviral pathway (Figure 2D) (H. Zhang et al. 2023).

The RSMV G protein acts as an effector, inducing rice autophagy via interaction with SnRK1B and phosphorylation of OsATG6b (a PI3K component), thereby activating ATG6‐mediated antiviral autophagy (Figure 2E). However, the virus exploits host autophagy to regulate G protein accumulation, preventing excessive toxicity and ensuring transmission (Huang et al. 2024). Infection cycle of RSMV was investigated in the vector *R. dorsalis* and the study revealed that RSMV initially accumulates in epithelial cells of the filter chamber and rapidly spreads to the salivary glands, midgut, hindgut, and central nervous system, significantly influencing feeding behaviour and extend the duration of nymphal development, which negatively affects population reproduction (P. Li et al. 2020; P. Zhao et al. 2019). Moreover, the M protein of RSMV interacts with the CCP functional domain of the *Hig* gene in *R. dorsalis*, potentially participating in vector antiviral processes (Q. Wang et al. 2010). Recent studies further demonstrated that mixed infections of RSMV with other viruses enhance its pathogenicity. For instance, Jia et al. found that co‐infection with RSMV and RGDV significantly promoted RSMV proliferation and transmission efficiency (D. Jia et al. 2022).

### 2.2 Maize Dwarf Mosaic Virus (MDMV)

MDMV belongs to the genus *Potyvirus* within the family *Potyviridae* and primarily infects maize, sorghum, and other grasses. It is widespread globally but has not yet been reported in China (Chen et al. 2023). MDMV is transmitted non‐persistently by aphids and can also spread via mechanical sap transmission and seeds. The MDMV genome is a positive‐sense single‐stranded RNA approximately 9500 nt long (Kannan et al. 2018) and encodes a single polyprotein a strategy used by potyviruses (Figure 1B). In the Kurdistan region of Iraq, natural MDMV infection in rice fields was detected for samples collected during 2020 and 2021, identifying rice as a new host. Aphid vectors, including *Rhopalosiphum maidis*, *R. padi*, and *Myzus persicae*, can transmit MDMV to rice, with higher transmission efficiency with *R. maidis* and *R. padi*. Infected rice plants show stripe and mosaic symptoms when aphid‐transmitted, while mechanical inoculation causes chlorosis and severe mosaic symptoms. MDMV has not been detected in rice seeds, suggesting low or no seed transmission efficiency (Haji et al. 2022).

### 2.3 Rice Tiller Inhibition Virus (RTIV)

A new insect‐borne polerovirus, RTIV, was identified via HTS in wild rice (*Oryza rufipogon*) from Guangxi University (Wenkai Yan et al. 2023). RTIV (*Solemoviridae*) has a 5914‐nt genome with seven ORFs, structurally resembling other poleroviruses (Figure 1C). Phylogenetic analysis revealed that RTIV is closely related to wheat leaf yellowing‐associated virus (WLYaV) and sugarcane yellow leaf virus (ScYLV). RTIV can be transmitted to cultivated rice (*O. sativa* L. spp. *japonica*, cv. Nipponbare) and *AGO18* knockout mutant (*ago18*) by the aphid *R. padi*. Infected rice plants show significant tillering reduction but no stunting or leaf discoloration. Transcriptome analysis revealed that RTIV infection alters the expression of tillering‐related genes, such as *CCD8B*, *ERG1* and *MOC1*. The virus appears to interfere with rice defence and tillering regulatory pathways, resulting in low tillering. The discovery of RTIV raises concerns about spillover risks from wild to cultivated rice, posing potential threats to rice production.

### 2.4 Rice Tiller Inhibition Virus 2 (RTIV2)

A new positive‐sense single‐stranded RNA virus identified through HTS in wild rice preserved in Guangxi University (L. J. Zhu et al. 2024). This virus was named RTIV2 due to the similar symptoms of inhibitng tillering as RTIV. RTIV2 belongs to the genus *Polerovirus* within the *Solemoviridae* family. The full genome length of RTIV2 is 5822 nucleotides, and its genomic structure is similar to other viruses in the same genus, containing seven ORFs (Figure 1B). Phylogenetic analysis indicates that RTIV2 is closely related to panicum distortion mosaic virus (PDMV). The P0 protein functions as a weak VSR. RTIV2 carries specific mutations in its VSR gene, reducing the viral ability to suppress RNA silencing. Co‐infection with umbraviruses pea enation mosaic virus 2 (PEMV2) or tobacco bushy top virus (TBTV) exhibits a synergistic effect. In the presence of PEMV2, RTIV2 can be mechanically transmitted to *Nicotiana benthamiana* (L. J. Zhu et al. 2024). Rice less tiller virus (RLTV) was identified from another wild rice plant preserved in Guangxi University, while rice dwarf polerovirus (RDPV) was identified from cultivated rice in paddy fields in Hainan, China (W. Yan et al. 2025; Yu et al. 2024). RDPV infected plants exhibit symptoms of dwarfism and reduced tillering. Currently, RDPV has already spread in Sanya City, Hainan (Yu et al. 2024). The transmission vectors of RDPV are the rice whitefly (*Bemisia formosana* Takahashi) and the aphid (*R. padi*), the only polerovirus reported transmitted by two completely different insect vectors. RLTV can be transmitted from wild rice to cultivated rice via *R. padi*, leading to reduced tillering symptoms in rice plants (W. Yan et al. 2025). RLTV and RDPV share 93.4% and 92.7% identities in nucleotide sequence to RTIV2, respectively, thus they are different isolates of the same virus.

### 2.5 Rice Virus A (RVA)

RVA was identified in rice plants displaying viral disease symptoms in Korea using HTS (F. Zhao et al. 2017). RVA belongs to the family *Tombusviridae* but has not been classified into any specific subgenus. RVA genome resembles those of *Tombusviruses* and *Zeaviruses*. However, phylogenetic analysis clusters RVA with *Aureusviruses*. Thus far, RVA is the largest virus in this family, with a genome length of 4832 nt and five ORFs: ORF1 encodes a 32‐kDa protein (p32). A readthrough of an amber stop codon forms a 90‐kDa protein (p90) encoding RdRp. ORF3 encodes a 49‐kDa capsid protein (p49). ORF4 encodes a 24‐kDa movement protein (MP). ORF5 encodes a 19‐kDa protein predicted to be a VSR (Figure 1D). RT‐PCR detection revealed an infection rate of 35.5% in Korean rice fields, suggesting that RVA has become endemic in Korea.

### 2.6 Rice Dwarf‐Associated Bunya‐Like Virus (RDaBV)

In 2019, dwarf rice plants with reduced tillering and unfilled grains was discovered in Zhejiang Province, China (D. Wang et al. 2022). RT‐PCR failed to detect any known viruses such as RBSDV, RDV, RGDV, RRSV, RSMV, RSV and SRBSDV. HTS analysis identified a new bunyavirus, named rice dwarf‐associated bunya‐like virus (RDaBV). RDaBV belongs to the order *Bunyavirales* and the family *Discoviridae*, with a genome composed of three segments (Figure 1E): L segment (6562 nt) encodes an RdRp with a conserved *bunya_RdRp* superfamily domain; M segment (1667 nt) encodes a nonstructural protein (NS), which potentially acts as a VSR and a determinant of disease symptoms; while S segment (1120 nt) encodes a nucleocapsid protein (N) on the complementary strand and a small nonstructural protein (NSs) on the positive strand, with the latter speculated to function as a movement protein. Phylogenetic analysis shows that RDaBV is closely related to viruses of *Discoviridae*. Preliminary studies indicate that common rice virus vectors (e.g., *Sogatella furcifera*, *Nilaparvata lugens*, *Laodelphax striatellus*, and *R. dorsalis*) cannot successfully transmit RDaBV. Instead, viruses of *Discoviridae* are typically transmitted by fungi, suggesting RDaBV might also be a fungal‐transmitted virus. RDaBV has spread widely in rice fields across Zhejiang, necessitating further evaluation to assess its potential threat to rice production.

### 2.7 Rice Latent Virus 1 and 2 (RLV1 and RLV2)

Two novel mastreviruses (Figure 1F), named rice latent virus 1 (RLV1) and RLV2, were identified in two undescribed AA‐genome wild rice species (*Oryza* sp. Taxon A and *Oryza* sp. Taxon B) from the Cape York Peninsula in Queensland, Australia (Kraberger et al. 2017). Both viruses belong to the genus *Mastrevirus* within the family *Geminiviridae*. Twenty‐two RLV1s were detected in both Taxon A and Taxon B and ranged in size from 2754 to 2758 nt, with > 98% sequence identities with one another. RLV2 was detected in only Taxon B and the genome was 2843 nt. Their genome structure resembles known mastreviruses, with the positive strand encoding capsid (V1) and movement (V2) proteins and the negative strand encoding replication‐associated proteins (C1 and C1:C2). Phylogenetic analysis showed that RLV1 is closely related to *eragrostis minor* streak virus (EMSV) and miscanthus streak virus (MiSV), while RLV2 is most closely related to *axonopus compressus* streak Virus (ACSV). No disease symptoms were observed in wild rice samples, suggesting a latent infection with RLV1 and RLV2. The discovery of RLV1 and RLV2 marks the first identification of mastreviruses in *Oryza* species.

### 2.8 Rice Curl Dwarf‐Associated Virus (RCDaV)

In 2017, rice plants exhibiting dwarfing and curling tillers in rice fields were discovered in Zhejiang Province, China. Using Dot‐ELISA and RT‐PCR, none of the eight reported rice viruses including RBSDV, RDV, RGDV, RGSV, RRSV, RSMV, RSV and SRBSDV was test positive (Tianze Zhang et al. 2021). Subsequent RNA‐seq and RT‐PCR analyses led to the discovery of a novel picornavirus, provisionally named rice curl dwarf‐associated virus (RCDaV). The genome of RCDaV is 8987 nt long and contains two major ORFs (Figure 1G). ORF1 (2409 nt) encodes a protein of 802 amino acids and ORF2 (5682 nt) encodes a protein of 1893 amino acids, with a 297 nt intergenic region (IGR) between them. Unlike the typical 3C protease cleavage site Q(E)/G(S) in most picornaviruses, the 3C protease (3Cpro) of RCDaV recognises a conserved EPT/S cleavage site, processing polyproteins into 12 mature proteins. Additionally, seven unclassified picornaviruses (maize‐associated picornavirus, MaPV; *Tetranychus urticae* associated picorna‐like virus 1, TUaPV1; aphis glycines virus 1, ApGlV1; cherry virus Trakiya, CVT; Hubei picorna‐like virus 51, HuPV51; Robinvale bee virus 6 isolate VN1‐8, RBV and *Leveillula taurica* associated picorna‐like virus 1, LtaPV) also use EPT/S as a cleavage site, suggesting evolutionary linkage. Phylogenetic analysis groups RCDaV and these unclassified viruses into a distinct branch.

### 2.9 Rice‐Associated Noda‐Like Virus 1 and 2 (RNLV1 and RNLV2)

Rice plants exhibiting dwarfing symptoms in Shanghai (2019) and Zhejiang (2016) were collected and two novel noda‐like viruses named rice‐associated noda‐like virus 1 (RNLV1) and rice‐associated noda‐like virus 2 (RNLV2) were identified using HTS (Xie et al. 2022). Both viruses belong to the family *Nodaviridae* and remain unclassified. The genomes consist of two positive‐sense single‐stranded RNAs. RNLV1 consists of RNA1 (3335 nt) and RNA2 (1769 nt), while RNLV2 consists of RNA1 (3279 nt) and RNA2 (1525 nt) (Figure 1H). Each virus encodes three ORFs: RdRp, a VSR and a capsid protein (CP). The CP of RNLV1 contains two Peptidase A21 domains and can self‐cleave under acidic conditions. RNLV1 replicates in insect cells (*Sf9*), while RNA1 replicates in plant cells. Phylogenetic analysis shows that RNLV1 and RNLV2 are distinct from other known noda‐like viruses, requiring further classification.

### 2.10 Multiple RNA Viruses Transmitted by Brown Planthopper

Highly resistant backcross lines of indica rice (Rby1 and Rby2) exhibited normal growth without *N. lugens* (brown planthopper) feeding and these rice lines only become infertile after being fed by *N. lugens* during the mature stage (Chao et al. 2021). Thirteen viruses were identified in these rice samples using HTS, including five known viruses (rice tombus‐like virus 1 [RTV1], RRSV, rice picorna‐like virus 1 [RPiV1], rice toti‐like virus 1 [RToV], and *Nilaparvata lugens* reovirus [NLRV]), along with eight novel viruses including three negative‐strand RNA viruses Fuyang mononega‐like virus (FMV), rice peribunya‐like virus (RPeV) and Fuyang phasma‐like virus (FPhV). and five positive‐strand RNA viruses Fuyang picorna‐like virus 2 (FPiV2), Fuyang picorna‐like virus 3 (FPiV3), Fuyang tombus‐like virus 2 (FTV2), Fuyang tombus‐like virus 3 (FTV3) and Fuyang noda‐like virus (FNV) (Figure 1I–K). RT‐PCR confirmed these viruses' presence, and small RNA analysis demonstrated active viral infections in sterile rice plants fed upon by brown planthoppers. The study suggested that these viruses were introduced into rice plants by brown planthopper feeding, as plants not exposed to planthoppers showed normal growth. This finding reinforces the role of brown planthoppers as significant vectors for these viruses.

## 3 Conclusion and Perspectives

**Figure 3:**
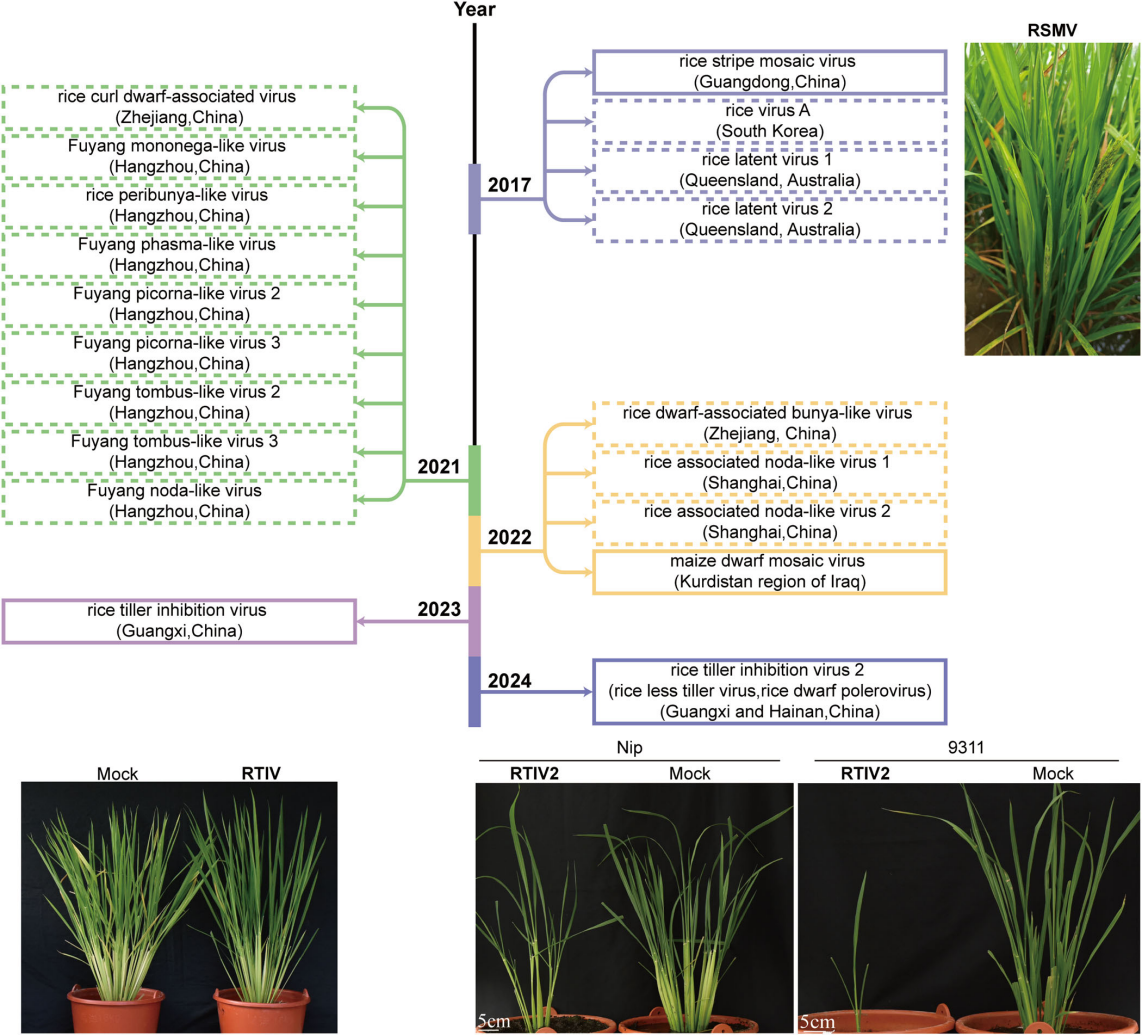
Timeline of emerging rice viruses discussed in this article. The viruses that have been experimentally validated following Koch's postulates are in indicated solid boxes, while those that have not are shown in the dashed boxes. Viruses reported in the same year are indicated by the same colour. Symptoms of RSMV, RITV and RTIV2 on rice are shown (courtesy of T. Zhang, W. Yan. and L‐J Zhu). [Color figure can be viewed at wileyonlinelibrary.com]

In the past decade, the rapid advancement of HTS technologies have led to the discovery of nearly 20 viruses in *Oryza* species, substantially deepening our understanding of the diversity, genome organisation, and evolutionary trajectories of rice viruses (Table 1 and Figure 3). Among them, a few viruses – such as RSMV, MDMV, RTIV, and RTIV2 – have been experimentally validated following Koch's postulates, whereas most remain in the exploratory phase, necessitating the construction of infectious clones and back‐inoculation assays to elucidate their biological characteristics and agricultural relevance (Figure 3; solid vs. dashed boxes).

**Table 1:** List of new viruses infecting *Oryza*.

Virus		Vector	Natural host	First report		Symptoms	Reference
Name	Classificiation			Location	year		
RSMV	*Cytorhabdovirus, Rhabdoviridae*	*Recilia dorsalis*, *Nephotettix virescen*s	*Oryza sativa*, *Digitaria sanguinalis*	Guangdong, China	2017	Slight dwarfing, leaves with striped mosaicism, increased tiller number, delayed heading, and mostly unfilled grains	Yang, Huang, et al.(2017)
MDMV	*Potyvirus*, *Potyviridae*	*Rhopalosiphum maidis*, *R. padi*, *Myzus persicae*	*O. sativa*, *Zea mays*, *Sorghum bicolour*, *S. halepense*	Kurdistan, Iraq	2022	Yellowing, streak and mosaic on foliage	Haji et al. (2022)
RTIV	*Polerovirus*, *Solemoviridae*	*R. padi*	*O. rufipogon*	Guangxi, China	2023	Low‐tillering disease in rice cultivar	Yan et al. (2023)
RTIV2	*Polerovirus*, *Solemoviridae*	Unknown	*O. rufipogon, O. sativa*	Guangxi & Hainan, China	2024	Stunting and reduced tillering	Zhu et al.(2024), Yu et al. (2024); Yan et al.(2025);
RVA	Unassigned, *Tombusviridae*	Unknown	*O. sativa*	South Korea	2017	Symptoms of viral disease, undescribed	Zhao et al. (2017)
RDaBV	Unassigned, *Discoviridae*	Unknown	*O. sativa*	Zhejiang, China	2022	Dwarfing, reduced tillering, and blighted grain	Wang et al. (2022)
RLV1	*Mastrevirus*, *Geminiviridae*	Unknown	*O*. sp. Taxon A, *O*. sp. Taxon B	Queensland, Australia	2017	No overt disease symptoms	Kraberger et al. (2017)
RLV2	*Mastrevirus*, *Geminiviridae*	Unknown	*O*. sp. Taxon B	Queensland, Australia	2017	No overt disease symptoms	Kraberger et al. (2017)
RCDaV	Unassigned, *Picornaviridae*	Unknown	*O*. *sativa*	Zhejiang, China	2021	Dwarfing and leaf curling	Zhang et al. (2021)
RNLV1	Unassigned, *Nodaviridae*	Unknown	*O*. *sativa*	Shanghai, China	2022	Dwarfing	Xie et al. (2022)
RNLV2	Unassigned, *Nodaviridae*	Unknown	*O*. *sativa*	Zhejiang, China	2022	Dwarfing	Xie et al. (2022)
FMV	*Mononegavirales*	*N. lugens*	*O*. *sativa* (Bph3‐carrying rice lines)	Hangzhou, China	2021	Unknown	Chao et al. (2021)
RPeV	*Peribunyaviridae*						
FPhV	*Phasmaviridae*						
FPiV2	*Picornaviridae*						
FPiV3	*Picornaviridae*						
FTV2	*Tombusviridae*						
FTV3	*Tombusviridae*						
FNV	*Nodaviridae*						

Abbreviations: FMV, Fuyang mononega‐like virus; FNV, Fuyang noda‐like virus; FPeV, rice peribunya‐like virus; FPhV, Fuyang phasma‐like virus; FPiV2, Fuyang picorna‐like virus 2; FPiV3, Fuyang picorna‐like virus 3; FTV2, Fuyang tombus‐like virus 2; FTV3, Fuyang tombus‐like virus 3; MDMV, maize dwarf mosaic virus; RCDaV, rice curl dwarf‐associated virus; RDaBV, rice dwarf‐associated bunya‐like virus; RLV1, rice latent virus 1; RLV2, rice latent virus 2; RNLV1, rice‐associated noda‐like virus 1; RNLV2, rice‐associated noda‐like virus 2; RSMV, rice stripe mosaic virus; RTIV, rice tiller inhibition virus; RTIV2, rice tiller inhibition virus 2; RVA, rice virus A.

These newly identified viruses display remarkable heterogeneity in genome architecture, vector adaptation, and transmission ecology, particularly within the *Picornaviridae*, *Nodaviridae*, and *Tombusviridae* families. Some may have originated from insects or possess atypical replication strategies, offering valuable insights into cross‐kingdom viral evolution.

While significant progress has been made in virus identification, preliminary functional studies, and vector associations, rice virology remains in a transitional phase—shifting from descriptive discovery to mechanistic research and breeding‐focused applications. A landmark study by Zhu et al. (Y. Zhu et al. 2025) analyzed 17 115 publicly available RNA‐seq datasets of Oryza species, revealing over 500 putative novel viruses alongside known pathogens. Although the infectivity and biological relevance of these viruses require experimental confirmation, this study underscores the vast, untapped diversity of rice‐associated viruses and suggests many more await discovery.

Moving forward, enhanced international collaboration will be critical to accelerate the characterisation of emerging rice viruses and translate these findings into actionable solutions for crop protection.

### 3.1 Commonalities and Divergences in Infection Strategies and Their Implications for Antiviral Breeding

Current findings not only highlight the complex viral threats rice cultivation faces under climate change and agricultural intensification, but also expose persistent gaps in our understanding of viral pathogenesis, ecological dissemination, and the development of deployable control strategies. Several viruses have been identified in wild rice, weeds, and arthropods, suggesting potential for cross‐species transmission. This underscores the need to reframe rice viral epidemiology from a tripartite perspective of virus–host–vector interactions (Chao et al. 2022; Haji et al. 2022; W. Jia et al. 2021).

On the one hand, many rice viruses utilise conserved mechanisms such as suppression of RNA silencing, modulation of phytohormone signalling (e.g., JA, GA and SA), and induction of autophagy‐mediated degradation to evade host immunity and facilitate replication. On the other hand, they also exhibit distinct pathogenic strategies. For instance, RSMV primarily suppresses host immunity through effector proteins, whereas RTIV‐like viruses impair rice tillering, representing a “developmental hijacking” model of disease. These shared and divergent mechanisms provide theoretical underpinnings for antiviral breeding: key host factors commonly targeted by viruses could serve as strategic entry points for broad‐spectrum resistance, while gene stacking and modular design approaches can be used to assemble multi‐layered defences against mechanistically diverse pathogens, thereby enhancing the breadth, durability, and ecological adaptability of resistant cultivars.

### 3.2 Current Research Hotspots and Key Future Directions

Research in rice virology is currently converging on several priority areas: (1) Functional validation and model system development for novel viruses: Establishment of infectious clones and *Agrobacterium*‐mediated delivery systems is essential for progressing from sequence discovery to mechanistic insights; (2) Mixed infections and co‐disease dynamics: Integration of single‐cell transcriptomics, tagged viruses, and co‐infection models is needed to elucidate cooperation, competition, and recombination among viruses; (3) Transmission ecology and host‐jump tracking: special focus is needed on potential spillover routes between wild rice, weeds, insect viruses, and cultivated rice to assess cross‐species transmission risks; (4) Immune network integration and core target identification: A systems‐level approach to RNA silencing, hormone signalling, and autophagy pathways can help construct interaction maps and pinpoint regulatory hubs; (5) Diversified antiviral breeding strategies: Based on the above targets, combined technologies – such as RNA interference, CRISPR/Cas genome editing, and synthetic transcription factors – should be employed to generate immune‐type rice lines with broad‐spectrum and long‐lasting resistance.

To improve the foresight and applicability of research outcomes, we recommend efforts be intensified in the following areas: (1) Innovation in virus detection and early warning technologies: Develop CRISPR‐based Diagnostics (CRISPR‐Dx) platforms, nanoprobe‐based sensors, and multiplex fluorescence systems for early and precise viral identification; (2) Systematic mining and utilisation of resistance resources: Screen wild relatives, landraces, and traditional cultivars for candidate resistance genes, and employ Quantitative Trait Locus (QTL) mapping and Genome‐Wide Association Studies (GWAS) to clone key loci; (3) Creation of resistant materials and varietal transformation: Use gene editing and molecular design breeding to engineer rice lines with durable, immune‐based resistance, facilitating the translation of research into breeding programmes; (4) Cross‐disciplinary integration and global collaborative platforms: Merge insights from plant virology, entomology, ecology, bioinformatics, and genetics to build shared international databases and coordinated management systems for rice virus threats.

### 3.3 Significance and Limitations of Current Research

Existing studies have systematically characterised the genomic traits, vector associations, and partial pathogenic mechanisms of emerging viruses, and have revealed that rice viruses can exploit RNA silencing suppression, hormone signalling manipulation, and autophagy modulation for successful infection. These advances offer new perspectives on viral evolution and virulence. However, most newly discovered viruses do not yet satisfy Koch's postulates and lack robust functional validation systems. Their host ranges, ecological adaptation, and field‐level transmission risks remain poorly defined, hindering accurate assessment of their agricultural impact. Furthermore, comparative studies on divergent infection strategies and ecological dynamics – including viral synergy, antagonism, and recombination – are still in their infancy. These gaps limit not only theoretical understanding but also the practical deployment of resistance resources and control measures.

In conclusion, rice virology is entering a stage of accelerated convergence – from pathogen discovery to mechanistic analysis, resistance design, and translational application. Against the backdrop of frequent viral emergence, climate‐driven transmission shifts, and the high intensification of agricultural systems, a shift from passive containment to proactive surveillance and genetic fortification is imperative. Future efforts should be guided by scientific foresight and interdisciplinary synergy to build an innovative, resilient, and domestically empowered rice viral disease prevention system. By targeting the development of broad‐spectrum, durable, and ecologically adapted resistance germplasm – alongside high‐throughput detection and predictive modelling – we move toward a closed‐loop pipeline of “accurate identification – real‐time surveillance – targeted intervention – molecular breeding.” This integrated approach will be essential for safeguarding global rice production and advancing China's leadership in agricultural biosecurity and technological self‐sufficiency.

## Conflicts of Interest

The authors declare no conflicts of interest.

## Data Availability

The authors have nothing to report.
